# From Mechanism to Management in Vasovagal Syncope: An Integrative Review

**DOI:** 10.3390/jcm15197360

**Published:** 2026-09-22

**Authors:** Sérgio Laranjo, Mário Martins Oliveira, Isabel Rocha

**Affiliations:** 1 Departamento de Fisiologia, NOVA Medical School (NMS), Faculdade de Ciências Médicas (FCM), Universidade NOVA de Lisboa, 1169-056 Lisbon, Portugal; sergiolaranjo@gmail.com; 2 Comprehensive Health Research Center (CHRC), NOVA Medical School, Faculdade de Ciências Médicas, NMS, FCM, Universidade NOVA de Lisboa, 1169-056 Lisbon, Portugal; 3 Serviço de Cardiologia Pediátrica, Hospital de Santa Marta, ULS de São José, 1169-024 Lisbon, Portugal; 4 Centro Cardiovascular da Universidade de Lisboa (CCUL), 1649-028 Lisbon, Portugal; 5 Arrhythmology Unit, Cardiology Department, Hospital de Santa Marta, Centro Hospitalar e Universitário de Lisboa Central, 1169-024 Lisbon, Portugal; m.martinsoliveira@gmail.com; 6 Institute of Physiology and Cardiovascular Autonomic Function Laboratory, Faculty of Medicine, Universidade de Lisboa, 1649-28 Lisbon, Portugal; 7 Medicine ULisboa for Health, Clinical Research and Innovation (MUHCRI), FMUL, Universidade de Lisboa, 1649-028 Lisbon, Portugal

**Keywords:** vasovagal syncope, reflex syncope, baroreflex, tilt testing, tilt training, pacing, cardioneuroablation, phenotype-guided therapy

## Abstract

**Background/Objectives:** Vasovagal syncope is the most common subtype of reflex syncope and a frequent cause of transient loss of consciousness. Although its prognosis is generally benign, recurrent episodes can cause injury, restrict daily activities, and impair quality of life; patients who remain symptomatic despite first-line measures present a therapeutic challenge. This review integrates current evidence on mechanisms, prediction, and treatment, examining whether arterial-pressure defence and baroreflex behaviour provide a useful physiological framework. **Methods:** We conducted a narrative integrative review of PubMed and ClinicalTrials.gov from inception to June 2026, prioritising randomised trials, meta-analyses, major observational studies, guidelines and multi-society statements. No formal risk-of-bias assessment or de novo quantitative synthesis was undertaken. **Results:** Current evidence supports a dynamic failure of cardiovascular compensation during orthostatic stress, with variable contributions from impaired vasoconstrictor support and cardioinhibition. Laboratory haemodynamic and autonomic markers may anticipate tilt-test outcome, but this should not be equated with prediction of spontaneous clinical recurrence. Tilt training may modify vasoconstrictor reserve and baroreflex indices, although clinical efficacy varies with protocol and adherence. Therapeutic options extend from counter-pressure manoeuvres and selected pharmacological agents to pacing in highly selected patients with documented clinically relevant asystole; cardioneuroablation remains an emerging intervention. **Conclusions:** The haemodynamic phenotype may inform treatment selection but should not be used as a stand-alone indication for invasive therapy. We propose an evidence-informed, mechanism-based model of care, incorporating patient-reported outcomes, shared decision-making and cautious evaluation of emerging predictive and interventional approaches.

## 1. Introduction

Reflex syncope comprises a heterogeneous group of neurally mediated transient loss-of-consciousness syndromes, including vasovagal syncope, situational syncope and carotid sinus syndrome [1,2] with a lifetime cumulative incidence of around a third of the population and a peak in adolescence and early adult life [3,4]. Vasovagal syncope is the most frequent subtype and constitutes the principal focus of this review. Although its prognosis is generally benign, recurrent episodes can cause injury, restrict driving and work, and impair quality of life [1,2]. For most patients, simple measures suffice, but a refractory minority continue to faint despite them, and it is in these patients that management has expanded most rapidly, with renewed evidence for selected pacing strategies and the emergence of catheter-based autonomic interventions [5]. This renewed interest has also been driven by advances in haemodynamic and autonomic phenotyping and by increasingly sophisticated approaches to physiological prediction.

The literature on vasovagal and reflex syncope is often compartmentalised, with pathophysiology, diagnosis, prediction and treatment reviewed separately. The purpose of this article is to draw these domains together while distinguishing established evidence from mechanistic interpretation and hypothesis-generating observations. We examine whether arterial-pressure defence and baroreflex behaviour provide a physiological thread linking susceptibility to a vasovagal response, laboratory prediction during tilt testing and response to non-invasive treatment. We then consider whether the haemodynamic phenotype can contribute to treatment selection, while emphasising that the phenotype alone is not an indication for invasive therapy. Evidence from the broader reflex syncope spectrum is included where it is mechanistically or clinically relevant.

## 2. Search Strategy

We conducted a narrative, integrative review of the literature. PubMed and ClinicalTrials.gov were searched from database inception to 30 June 2026. Search terms combined “reflex syncope” or “vasovagal syncope” with terms related to pathophysiology, diagnosis, prediction, and treatment, including “baroreflex”, “tilt test” or “head-up tilt testing”, “prediction”, “tilt training” or “orthostatic training”, “counter-pressure manoeuvres”, “midodrine”, “fludrocortisone”, “beta-blocker” or “metoprolol”, “cardiac pacing”, “closed-loop stimulation”, and “cardioneuroablation”. This review was restricted to publications in English. Reference lists of relevant reviews, guidelines and key original studies were also examined to identify additional seminal publications. Priority was given to randomised controlled trials, systematic reviews and meta-analyses, major observational studies, clinical guidelines, multi-society statements and ongoing registered trials. Older studies were included when important for the historical development or physiological interpretation of the field. ClinicalTrials.gov was searched for ongoing or recently completed studies on pacing, cardioneuroablation and other mechanism-based interventions. The literature search was performed independently by two authors, S.L. and I.R., and the selection and interpretation of the most relevant evidence were discussed by all authors. This was a narrative integrative review rather than a systematic review; no formal risk-of-bias assessment or de novo quantitative evidence synthesis was undertaken. Numerical effect estimates reproduced in the text or figures were taken directly from the cited publications and were not pooled across interventions by the authors. Conceptual diagrams were developed by the authors to synthesise the physiological and clinical relationships described in the text.

## 3. Clinical Burden and Physiological Foundations

### 3.1. Burden and Prognosis

In the Framingham cohort, the incidence of a first syncope of any cause was about 6 per 1000 person-years, and vasovagal syncope, the largest single category, carried no excess mortality [6]. The benign prognosis, however, sits alongside a heavy burden of morbidity: falls and fractures, road and occupational restriction, anticipatory anxiety, and a measurable loss of quality of life, the last increasingly recognised as a treatment target in its own right [1,2]. Because the condition is common, even a small absolute risk of injury translates into a large public-health footprint, and because it is benign, the threshold for invasive treatment must be set by symptom burden and quality of life rather than by mortality.

### 3.2. Pathophysiology: A Dynamic Failure to Defend Arterial Pressure

The vasovagal response can be understood as a dynamic failure of the mechanisms that normally maintain arterial pressure during orthostatic stress (Figure 1) [7,8]. Current concepts suggest that reflex syncope is not caused by a single trigger or pathway. Instead, it results from the interaction of cardiovascular, autonomic, humoral, respiratory and cerebral mechanisms that change over time [9].

Upon standing, gravity causes blood to pool in the dependent venous circulation, reducing venous return, cardiac filling and stroke volume. In healthy subjects, these changes are compensated by baroreflex-mediated sympathetic activation, peripheral and splanchnic vasoconstriction, and an increase in heart rate. In susceptible subjects, however, this compensation may become insufficient or unstable.

Direct recordings before syncope show that the vasovagal response develops over time rather than as a single abrupt reflex. During the first phase, compensation may include marked vasoconstriction and reduced cardiac vagal activity, followed by tachycardia and increasing haemodynamic instability, with changes in arterial pressure and heart rate. In the final phase, loss or withdrawal of sympathetic vasoconstrictor support may contribute to vasodilatation, while an increase in vagal activity may cause bradycardia. The relative contribution of these mechanisms varies between patients [10]. When cerebral perfusion becomes insufficient, consciousness is transiently lost [11,12].

The relative contribution of cardiac output and peripheral vascular resistance to the fall in arterial pressure has been debated for many years. Haemodynamic studies suggest that a progressive reduction in cardiac output, mainly due to reduced venous return and stroke volume, may be a major cause of hypotension during vasovagal syncope [13,14,15]. The contribution of systemic vascular resistance appears to be more variable and may differ according to age and the haemodynamic phenotype [16]. More recent direct measurements of regional blood flow and sympathetic nerve activity have also shown the importance of the interaction among venous return, regional vascular responses and sympathetic control [17]. Vasodilatation is, therefore, an important part of the vasovagal response, but it may not be the main mechanism in every patient.

Autonomic regulation also changes during the vasovagal response. Apparently conflicting findings of sympathetic activation and sympathetic withdrawal may reflect different phases of the same event. Early sympathetic activation may help to maintain arterial pressure, whereas later, this response may become insufficient or decrease [15,18,19]. Recent reviews also support a phase-dependent interaction between sympathetic and parasympathetic activity, rather than a simple switch from sympathetic to vagal dominance [20]. Cardiac vagal activation becomes particularly important during the final cardioinhibitory phase and may lead to sinus slowing, atrioventricular block or, in some patients, prolonged asystole.

The arterial baroreflex is an important link between these vascular and cardiac responses. It can be assessed non-invasively from the spontaneous relation between beat-to-beat arterial pressure and cardiac intervals, using sequence and frequency-domain methods [21,22]. However, studies of baroreflex sensitivity in vasovagal syncope have produced different results, with reduced, preserved and even increased sensitivity reported in different populations [19,23,24,25].

One possible reason for these differences is that baroreflex function is often described only by its sensitivity or gain. Other aspects are also important, such as its effectiveness—the proportion of spontaneous arterial-pressure changes that produce an appropriate reflex response—and the timing of this response [22,26,27]. A reflex with an apparently preserved gain may still be inadequate if it is activated inconsistently or responds too slowly to rapid changes in arterial pressure. Reduced baroreflex sensitivity and effectiveness have been reported immediately before tilt-induced syncope, while a longer baroreflex latency has been associated with poor orthostatic tolerance [24,25,27]. More recent studies using direct sympathetic recordings also support a progressive deterioration of baroreflex control before syncope [28].

The timing of these changes is important because the cardiovascular response during head-up tilt is highly non-stationary. Haemodynamic and autonomic conditions can change within a few minutes, particularly before syncope. Analyses based on long and relatively stable periods may therefore miss rapid changes in autonomic and baroreflex control and may partly explain differences between previous studies [29,30,31].

The capacity of the resistance vessels to compensate for reduced cardiac filling is another important part of arterial-pressure defence. Vasoconstrictor reserve therefore complements the assessment of baroreflex function: the baroreflex reflects the regulatory response, while vasoconstrictor reserve reflects the ability of the vascular system to produce an effective haemodynamic response [32,33]. Reduced vasoconstrictor reserve may be particularly important in vasodepressor and mixed responses.

The balance between vasodepression and cardioinhibition differs among individuals and may also vary between episodes in the same patient. This variability is the basis of the classical haemodynamic phenotypes of vasodepressor, cardioinhibitory and mixed responses [34]. This distinction is clinically relevant because different treatments act on different parts of the reflex.

The historical ventricular or Bezold–Jarisch hypothesis proposed that vigorous contraction of an underfilled ventricle activates cardiac mechanoreceptors and triggers the vasovagal response. This model is no longer sufficient to explain the full clinical spectrum of vasovagal syncope [35]. Vasovagal syncope has also been described in heart-transplant recipients despite cardiac denervation, showing that an intact cardiac afferent pathway is not essential for the response. In these observations, hypotension was associated with an abrupt withdrawal of sympathetic vasoconstrictor activity [36]. Current concepts, therefore, favour a more distributed mechanism involving central autonomic integration, cardiovascular and vascular responses, humoral factors, and cerebral regulation [9]. Functional imaging during orthostatic stress has also started to identify the central autonomic network involved in this response [37,38], supporting the view that syncope results from interactions between the brain, heart, and circulation, rather than from a single peripheral trigger.

Cerebral haemodynamics may also contribute directly to the transition from presyncope to loss of consciousness. Cerebral hypoperfusion has traditionally been considered mainly a consequence of systemic hypotension, but transcranial Doppler studies suggest that cerebral blood-flow velocity may start to fall before a major decrease in systemic arterial pressure in some patients. Recent studies have proposed a possible vasovagal syncope phenotype associated with impaired dynamic cerebral autoregulation [39]. One study described “primary cerebral autoregulation dysfunction” as a possible distinct subtype of vasovagal syncope [40]. These findings are interesting but still preliminary, and need independent prospective confirmation. They suggest that abnormal cerebrovascular regulation may contribute to symptoms before, or partly independently of, marked systemic hypotension in some patients.

Respiratory changes may also influence cerebral perfusion. Hyperventilation and hypocapnia may occur during orthostatic stress and presyncope, and the resulting cerebral vasoconstriction may further reduce cerebral blood flow. Orthostatic tolerance may, therefore, depend partly on the interaction among ventilation, arterial CO_2_, autonomic regulation and cerebral haemodynamics [41]. This may help to explain why cerebral symptoms and changes in systemic arterial pressure do not always develop in parallel.

Humoral mechanisms may also influence individual susceptibility. Adenosine has received particular attention in patients with abrupt syncope, little or no prodrome, and marked bradycardia or paroxysmal atrioventricular block, suggesting that different biochemical phenotypes may exist. Serotonergic pathways have also been proposed as modulators of central and autonomic responses, although their precise role in typical vasovagal syncope remains uncertain [42]. These observations again support the view that vasovagal syncope is heterogeneous rather than the result of one universal reflex mechanism.

A genetic contribution is also likely. Familial aggregation and twin studies support an inherited component of syncope susceptibility, although the mechanisms linking genetic predisposition with a specific autonomic or haemodynamic phenotype remain unclear [43]. At present, genetic background is probably better considered as a factor that modifies susceptibility rather than as an independent mechanism of the vasovagal response.

Taken together, current evidence supports a dynamic and distributed model of vasovagal syncope. Reduced cardiac filling challenges arterial-pressure defence; sympathetic and vascular compensation may initially be activated but later become insufficient or unstable; baroreflex gain, effectiveness and timing may deteriorate; and variable cardioinhibition may further reduce arterial pressure. Cerebral autoregulation, respiratory CO_2_ interactions, central autonomic integration and humoral influences may also affect the final transition to cerebral hypoperfusion and loss of consciousness. These mechanisms should not be considered as a fixed sequence occurring in every patient. Their relative contribution and timing may differ between individuals and may also vary between episodes in the same patient. This framework provides the physiological basis for the phenotyping, prediction and mechanism-informed treatment approaches discussed in the following sections.

Two components of this framework are particularly suitable for physiological assessment: baroreflex behaviour and vasoconstrictor reserve. These measures provide the basis for the haemodynamic phenotyping and laboratory prediction approaches discussed in the following section.

## 4. Diagnosis, Phenotyping and Prediction

### 4.1. Diagnosis and Haemodynamic Phenotyping

The diagnosis rests first on the history, which, in typical cases, is sufficient [1]. Where it is not, head-up tilt testing reproduces the reflex under observation and classifies the haemodynamic response; the standardised, nitrate-potentiated protocols developed in the 1990s remain the workhorse [44] (Figure 2). The tilt test does not establish the cause of spontaneous episodes so much as confirm a susceptibility and define its haemodynamic type. For recurrent, unexplained, or infrequent episodes, the implantable loop recorder captures the rhythm during a spontaneous event and has been shown to allow effective, mechanism-specific therapy, in particular by documenting the asystole that selects candidates for pacing [45]. The haemodynamic classification that emerges from tilt testing, cardioinhibitory, vasodepressor, or mixed, is central to the phenotype-guided approach proposed below [34]. The test has well-known limits, imperfect reproducibility, and false positives in healthy people, so a positive result confirms a susceptibility rather than proving the cause of a given spell; the main differential diagnoses, orthostatic hypotension, postural tachycardia syndrome, and arrhythmic (cardiac) syncope, are separated by the history, the electrocardiogram and, where needed, prolonged rhythm monitoring [1,2].

### 4.2. Prediction of Tilt-Test Outcome

Several physiological approaches can anticipate whether syncope will occur during an ongoing tilt test. Early studies showed that asymptomatic systolic-pressure reductions or pressure oscillations in the first minutes can foreshadow a positive response [46], and an algorithm combining the trend and variability in heart rate and blood pressure predicted tilt-induced syncope in more than a thousand patients with high sensitivity and specificity, providing several minutes of warning [47]. Machine-learning analyses of tilt-test signals are beginning to automate this laboratory prediction, although they remain at the proof-of-concept stage [48]. Preliminary unpublished observations from our group suggest that baroreflex timing and related autonomic measures may provide additional mechanistic information [49,50], but these data are not used here as quantitative evidence and require independent validation. Importantly, prediction of tilt-test positivity or time-to-syncope during controlled orthostatic stress should not be equated with prediction of spontaneous clinical recurrence in everyday life (Figure 3).

## 5. Treatment: A Stepped, Mechanism-Led Approach

Treatment may be organised as a stepped pathway in which the intensity and invasiveness of intervention rise with symptom burden. The haemodynamic phenotype may inform that pathway, but invasive treatment requires additional clinical and mechanistic selection criteria (Table 1 and Table 2, Figure 4).

### 5.1. Education, Lifestyle Measures and Counter-Pressure Manoeuvres

All patients should receive education about the mechanism and the benign nature of the faint, instruction in recognising and avoiding triggers, advice on liberal salt and fluid intake, and training in physical counter-pressure manoeuvres, which abort or postpone the faint by recruiting muscle and raising pressure and which reduced recurrence in a randomised trial [51]. These measures are effective, safe and first-line [1,2].

### 5.2. Pharmacotherapy

The pharmacological evidence is modest and selective. Metoprolol, a β1-selective beta-blocker, was ineffective overall in the original POST trial [52]. Earlier subgroup analyses raised the possibility of benefit in older patients, but this hypothesis was directly tested in the 2026 POST5 randomised placebo-controlled trial of patients aged ≥40 years and was not confirmed: syncope recurred in 31% of patients receiving metoprolol and 40% receiving placebo (*p* = 0.69) [58]. In the accompanying individual-patient-data meta-analysis of four randomised trials, recurrence occurred in 34% of beta-blocker-treated patients and 34% of placebo-treated patients (odds ratio 0.99, 95% confidence interval 0.43–2.29) [58]. The current evidence, therefore, does not support an age-dependent benefit of beta-blocker therapy. Fludrocortisone gave a borderline reduction in recurrence [53], whereas midodrine, an alpha-agonist that supports vascular tone, reduced recurrence in selected patients [54]. A meta-analysis separating blinded from unblinded trials similarly found that midodrine and selective serotonin reuptake inhibitors, but not beta-blockers or fludrocortisone, separated from placebo under blinding [57].

### 5.3. Tilt (Orthostatic) Training

Repeated, prolonged exposure to upright posture was proposed as a non-invasive treatment that might habituate the orthostatic reflex [68,69]. Its evidence base has been contested: a meta-analysis restricted to randomised trials found no significant effect, a result attributed in part to heterogeneous protocols and poor adherence to unsupervised home training [55]; a randomised trial confined to older patients was likewise neutral [70]. A more recent and larger meta-analysis reached the opposite conclusion, finding that tilt training, like counter-pressure manoeuvres, significantly reduces recurrence [56]. This discrepancy may partly reflect differences in training intensity and adherence. Physiological studies suggest that training can shift the orthostatic response from greater reliance on tachycardia towards greater reliance on vasoconstriction, increase vasoconstrictor reserve, and improve indices of baroreflex function [71,72,73]. Preliminary unpublished observations from our group are consistent with an adherence-dependent clinical effect [74] but are not used here as quantitative efficacy evidence. Tilt training is non-invasive, reversible and inexpensive; however, its long-term clinical efficacy is heterogeneous, and adherence remains a major limitation. The available evidence, therefore, supports a physiological hypothesis linking arterial-pressure defence, tilt-test susceptibility and adaptation to structured orthostatic training, rather than a validated predictive-treatment algorithm (Figure 5).

### 5.4. Cardiac Pacing and Closed-Loop Stimulation

Cardiac pacing has the most instructive history in the field, and it cautions against simple conclusions (Figure 6). Early unblinded trials were strikingly positive: in the Vasovagal Syncope International Study, dual-chamber pacing cut recurrence to 5% against 61% with no device [75]. When the comparison was made double-blind, by implanting every patient and randomising the pacemaker on or off, the benefit evaporated, for neither the Second Vasovagal Pacemaker Study nor the SYNPACE trial found an advantage of active pacing [76,77]. The lesson is that much of the early benefit was an expectation and a surgical effect, and that pacing helps only when two conditions are met: a documented bradycardia or asystole as the mechanism, and an algorithm that intervenes early enough to matter.

Selecting patients by mechanism revived pacing, but a positive or cardioinhibitory tilt response is not, by itself, an indication for implantation. Current ESC pacing guidance recommends pacing only in highly selected patients aged >40 years (mostly >60 years) with severe, recurrent, and unpredictable reflex syncope, particularly when episodes carry a high risk of injury or occur with little or no prodrome, and when clinically relevant asystole is documented spontaneously, during carotid sinus massage, or during tilt testing [67]. ISSUE-3 demonstrated benefit when spontaneous asystole was documented by an implantable loop recorder [59]. More recent evidence from SPAIN and BioSync CLS supports closed-loop stimulation in selected patients with severe recurrent syncope and tilt-induced asystole [60,61,78]. Closed-loop stimulation may act earlier than conventional rate-drop pacing by sensing changes in myocardial contractility during the initial sympathetic phase [79,80], but its efficacy should not be extrapolated to unselected tilt-positive patients.

The balance between cardioinhibition and hypotensive susceptibility is central to patient selection. A pacemaker cannot directly restore peripheral vasoconstriction, and a dominant vasodepressor response may, therefore, limit protection even when bradycardia or asystole is treated. Tilt testing can be useful not only to document an asystolic response but also to identify associated hypotensive susceptibility, which should be addressed with non-device measures when present [67]. Overall, pacing, including closed-loop stimulation, is effective in appropriately selected patients with severe recurrent reflex syncope and documented clinically relevant cardioinhibition or asystole; its value in a purely vasodepressor phenotype remains unproven, and the lifelong device burden is an important consideration, particularly in younger patients (Table 2).

### 5.5. Cardioneuroablation

Catheter ablation of atrial ganglionated plexi, cardioneuroablation, aims to reduce the excessive cardiac vagal influence underlying cardioinhibitory responses and has emerged rapidly as a potential therapeutic option [81,82]. The first randomised efficacy trial reported syncope in 8% of ablated patients versus 54% of controls at two years [62]; however, this study was open-label and did not include a sham procedure. This limitation is particularly relevant in reflex syncope, in which the early pacing literature demonstrated a marked attenuation of apparent treatment benefit when blinding was introduced. A further randomised trial compared ablation strategies rather than ablation against sham or established therapy [65], while meta-analyses report low recurrence rates but are dominated by observational studies [63,64,83] (Figure 7).

A 2024 multi-society scientific statement set out patient selection, technique and evidence gaps, while emphasising that most data remain observational and that the long-term consequences of permanent vagal denervation are uncertain [66]. Cardioneuroablation should, therefore, be regarded as an emerging intervention for carefully selected patients with severe recurrent cardioinhibitory reflex syncope, rather than as an established alternative equivalent to pacing. The absence of sham-controlled efficacy trials, uncertainty regarding procedural standardisation and endpoints, and limited long-term outcome data should be discussed explicitly during shared decision-making. Its place relative to pacing, non-invasive treatment and no intervention remains the subject of ongoing trials (Table 3).

These developments illustrate how the treatment of reflex syncope has evolved over the past four decades, from non-invasive orthostatic training and the reassessment of cardiac pacing to the emergence of cardioneuroablation and the comparative trials now under way (Figure 8).

## 6. Towards Phenotype-Guided, Patient-Centred Care

### 6.1. A Phenotype-Guided Hypothesis

The haemodynamic classification of the tilt response, cardioinhibitory, vasodepressor or mixed, remains clinically informative [34,84], but it should be considered as one component of a broader selection process rather than as a treatment algorithm. Therapies act on different parts of the reflex: pressor drugs, counter-pressure manoeuvres, and tilt training primarily address hypotensive susceptibility, whereas pacing targets clinically relevant bradycardia or asystole and cardioneuroablation targets cardiac vagal mechanisms. A documented cardioinhibitory or asystolic response may, therefore, support consideration of an invasive strategy only when accompanied by an appropriate clinical profile, including severe recurrent and unpredictable events, injury risk or short/absent prodrome, age and lifetime treatment burden, and assessment of the associated hypotensive component [67]. A pure vasodepressor response points instead towards pressor and training strategies, while mixed responses require attention to both components (Table 4, Figure 4). This remains a hypothesis-generating framework rather than a validated treatment algorithm.

There is a physiological rationale for expecting haemodynamic phenotypes to differ at the level of autonomic and baroreflex control. Baroreflex sensitivity during tilt has been reported to differ by response type, with predominant impairment in the vasodepressor form and instability in the cardioinhibitory form [85,86]. Preliminary unpublished observations from our group suggest that the early autonomic signature may also vary across phenotypes [50], but these observations are not presented as established evidence. Whether distinct autonomic substrates underlie haemodynamic phenotypes, and whether matching treatment to substrate improves spontaneous clinical outcomes, has not been tested directly. Prospective cohorts characterised mechanistically and stratified by phenotype will be required to address this question.

### 6.2. The Patient’s Experience: Quality of Life and Daily Living

Because the disability of reflex syncope is driven as much by the fear of fainting as by the faints themselves, the patient’s experience is an essential outcome.

Emotional stress, fear, and anxiety may also act as triggers or modulators of the vasovagal reflex in susceptible individuals, particularly in emotionally triggered vasovagal syncope, through stronger central autonomic influences on cardiovascular reflex control [1,87]. The relationship is likely to be bidirectional: recurrent and unpredictable syncope may itself generate anticipatory anxiety and avoidance behaviour [87]. Anxiety should, therefore, be regarded as a potential trigger and consequence of vasovagal syncope rather than as an established primary cause.

Validated, syncope-specific instruments capture the worry, the activity restriction, and the social impact that matter to patients [88], and treatments that reduce events also improve these domains, whether the intervention is tilt training or cardioneuroablation [74,83]. In some patients, this psychological burden may become clinically prominent, and psychogenic pseudosyncope—attacks that resemble fainting but occur without a corresponding fall in blood pressure—may coexist with or mimic the vasovagal reflex and should be recognised, as it requires psychological rather than cardiovascular management [1,89]. The practical consequences, restrictions on driving and on some occupations in particular, weigh heavily on younger patients and should be addressed explicitly within the guideline framework [1]. Assessment of quality of life, and shared decision-making informed by it, should accompany every step of the pathway.

### 6.3. Organising Care: Syncope Units, Value and the Patient’s Voice

How care is organised matters as much as which treatment is chosen. The fragmented assessment of transient loss of consciousness, repeated across emergency departments and specialities, is inefficient and low-yield, and the response has been to concentrate it in a dedicated syncope unit, in effect an integrated practice unit for the condition [90]. The evidence that this works is consistent (Figure 9). The randomised Syncope Evaluation in the Emergency Department Study raised the diagnostic yield from 10% to 67% and cut admissions from 98% to 43%, without harming outcomes [91]; a strict, guideline-based pathway applied across general hospitals reached a diagnosis in almost all patients, while admitting only a quarter [92]; and a European position statement, endorsed by the Heart Rhythm Society, set out the rationale, the staffing and the facilities such a unit requires [90,93]; a parallel position statement from the European Federation of Autonomic Societies set out an analogous model for autonomic units more broadly [94]. In practice, the syncope unit provides an organisational setting in which the stepped, phenotype-guided pathway proposed above could be implemented.

The evidence that this approach improves diagnostic yield and reduces hospital admissions is summarised in Table 5.

This organisation aligns naturally with value-based healthcare, in which the outcomes that matter to patients are weighed against the cost of achieving them. For a benign but disabling condition, those outcomes are not mortality, but the frequency of events, the burden of injury and, above all, the patient’s own experience. Patient-reported outcome measures capture this: a validated, syncope-specific instrument records the worry, the activity restriction, and the social impact of fainting [88], and the wider movement towards standardised outcome sets in cardiology, exemplified by the consensus minimum set for atrial fibrillation, offers a template that syncope still lacks [95,96].

Patient-reported experience measures, which assess the process of care rather than its result, complete the picture. Embedding both within the syncope unit would let services benchmark not only their diagnostic yield and cost but the quality of life and the experience they deliver, and would give the shared decisions that run through the pathway a measurable anchor. The integrative scope of the present review is also one of its principal strengths. Compared with previous reviews that have often addressed pathophysiology, diagnosis, or treatment separately, we aim to bring autonomic and haemodynamic mechanisms, phenotyping, prediction, and treatment together within a single mechanism-informed framework. Its principal limitations derive from its narrative design: the study selection was not based on a systematic-review protocol, no formal risk-of-bias assessment or de novo quantitative synthesis was performed, and several proposed links between the physiological phenotype and treatment response remain hypothesis-generating and require prospective validation.

## 7. Emerging Directions and Future Perspectives

### 7.1. Digital and Intelligent Tools

Artificial intelligence is increasingly being explored in the assessment and management of syncope. Machine-learning models can classify tilt-test responses and estimate the likelihood of fainting from physiological signals recorded during controlled testing [48,97]. These results should be regarded as proof-of-concept rather than evidence of validated real-world clinical utility. In a large emergency-department cohort, machine-learning models did not outperform a well-derived clinical risk score, illustrating the importance of external validation, calibration, and realistic comparison with standard clinical approaches [98]. A multidisciplinary statement has identified potential roles for artificial intelligence in event classification, diagnosis, recurrence-risk assessment, and disposition decisions, while emphasising validation, transparency, and equity [99].

Wearable technologies are particularly promising but remain investigational. A consumer smartwatch using photoplethysmographic heart-rate variability and machine learning predicted vasovagal syncope during laboratory tilt testing with an area under the curve of about 0.91 and several minutes of warning [100]. This demonstrates promising proof-of-concept performance during provoked syncope, but it does not establish the prospective prediction of spontaneous events in ambulatory life. External validation in independent populations, assessment under real-world conditions, and demonstration that alerts are timely and specific enough to improve clinically relevant outcomes are required before such systems can be considered clinically established. Large language models may also assist with extraction of clinical events from free-text records [101], but their use in syncope care likewise requires prospective validation and should remain adjunctive to clinical judgement.

### 7.2. Emerging and Disruptive Directions

Beyond the baroreflex, a second messenger is redefining part of the field. Some patients faint suddenly, without the usual prodromes and with a structurally normal heart, and these patients have strikingly low plasma adenosine and a distinct receptor profile, a low-adenosine phenotype that behaves as a purinergic mirror-image of classic vasovagal syncope and that tends to produce abrupt asystole from paroxysmal atrioventricular block [102]. The discovery matters because it is treatable on its own terms: the adenosine antagonist theophylline roughly halved recurrence in these patients in a matched study verified by an implantable monitor, the benefit concentrated in the asystolic form [103]. This example illustrates the type of mechanism-based precision that has largely been lacking in the field.

Diagnosis is being personalised in parallel. Tilt-table testing, long applied as a single fixed procedure, is better tailored to the individual: a meta-regression of nearly a hundred studies shows that its yield depends on the patient’s age and on the provocation used, with nitroglycerin and isoproterenol raising sensitivity at some cost to specificity, which supports age-adjusted, provocation-augmented, and shortened protocols, rather than one universal test [104].

Two additional areas are beginning to draw attention. Autonomic neuromodulation is being miniaturised and made non-invasive: transcutaneous stimulation of the auricular vagus nerve has improved orthostatic intolerance and autonomic balance in a sham-controlled trial in the related postural tachycardia syndrome, and although it has not yet been tested specifically in reflex syncope, it points to a reversible, wearable alternative to ablation [105]. And the predisposition is, in part, inherited: the first genome-wide association study on syncope identified a locus on chromosome 2, near a gene most strongly expressed in the brain, supporting a heritable and centrally mediated susceptibility and opening the door, eventually, to a risk that can be read from the genome [106].

### 7.3. Priorities for Future Research

Several clinically relevant questions can now be addressed. Head-to-head trials are comparing pacing with cardioneuroablation for selected cardioinhibitory patients, and registries are gathering longer-term and real-world data that the observational literature lacks (Table 3). The durability of cardioneuroablation, the consequences of permanent vagal denervation, and the identification of patients most likely to benefit remain priorities. For prediction, machine-learning and wearable approaches should move from laboratory discrimination of tilt-test outcome to external validation and prospective prediction of spontaneous clinical events [48,99,100]. For mechanism-informed care, the central task is to test directly whether physiological markers and the haemodynamic phenotype improve treatment selection or monitoring beyond standard clinical criteria. The routine measurement of patient-reported outcomes within syncope units would also allow treatments to be judged against outcomes that matter most to patients [90,95].

## 8. Conclusions

Vasovagal syncope is common, generally benign and, when recurrent, potentially disabling. The current evidence supports a dynamic failure of arterial-pressure defence with variable contributions from vasodepression and cardioinhibition; the haemodynamic phenotype may inform interpretation and treatment, but invasive therapy requires additional clinical and mechanistic selection criteria. Laboratory prediction, baroreflex-based phenotyping, cardioneuroablation and wearable/AI approaches remain promising areas in which prospective validation against spontaneous clinical outcomes is still required.

## Figures and Tables

**Figure 1:**
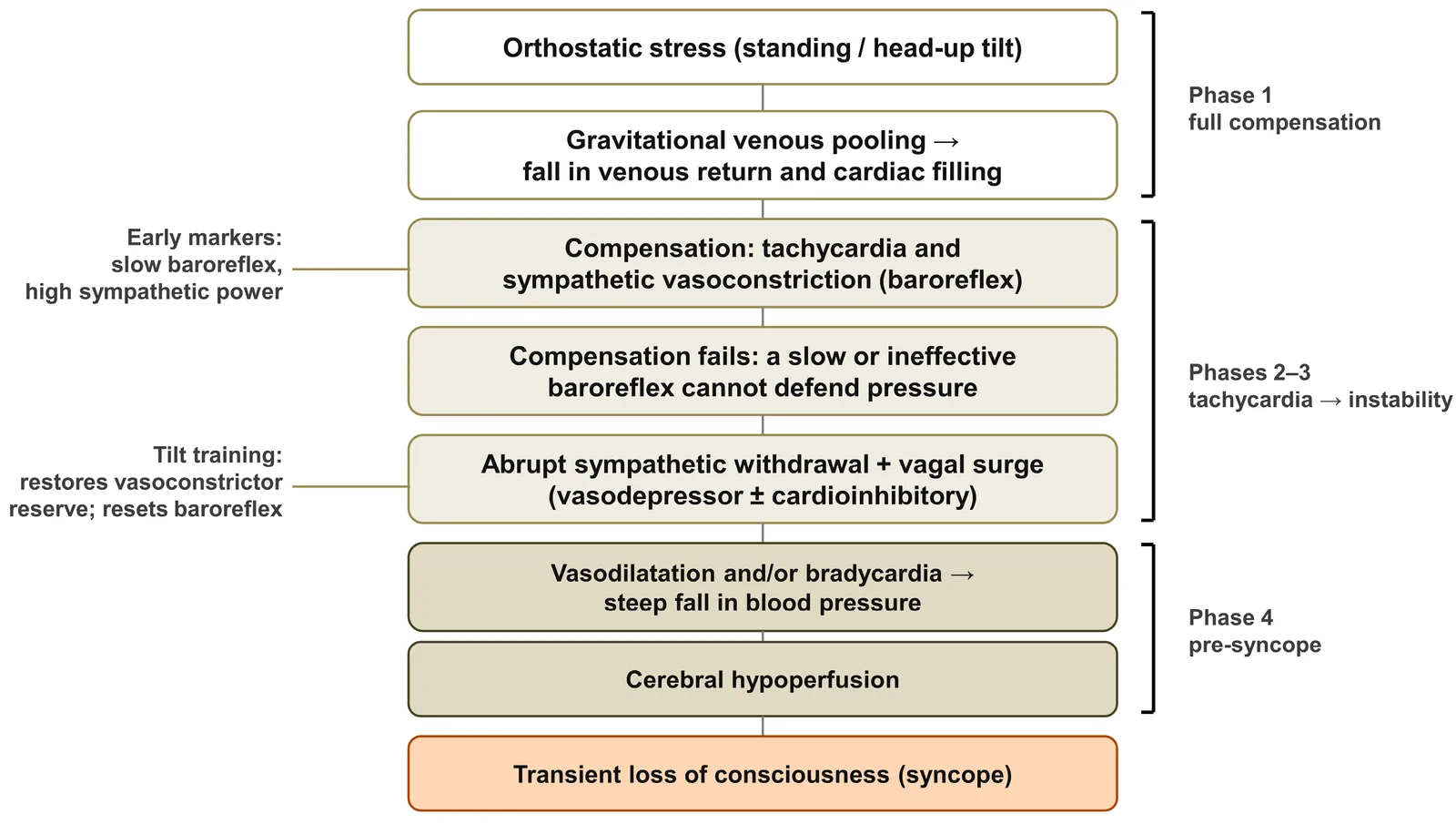
The pathophysiology of the vasovagal response. Orthostatic stress causes venous pooling and a fall in cardiac filling; when a slow or ineffective baroreflex cannot sustain the compensatory vasoconstriction, sympathetic withdrawal and a vagal surge produce vasodilatation and bradycardia, a steep fall in pressure, and cerebral hypoperfusion. The bracket on the right shows the four haemodynamic phases described before fainting; the annotations on the left mark where the early markers and tilt training act.

**Figure 2:**
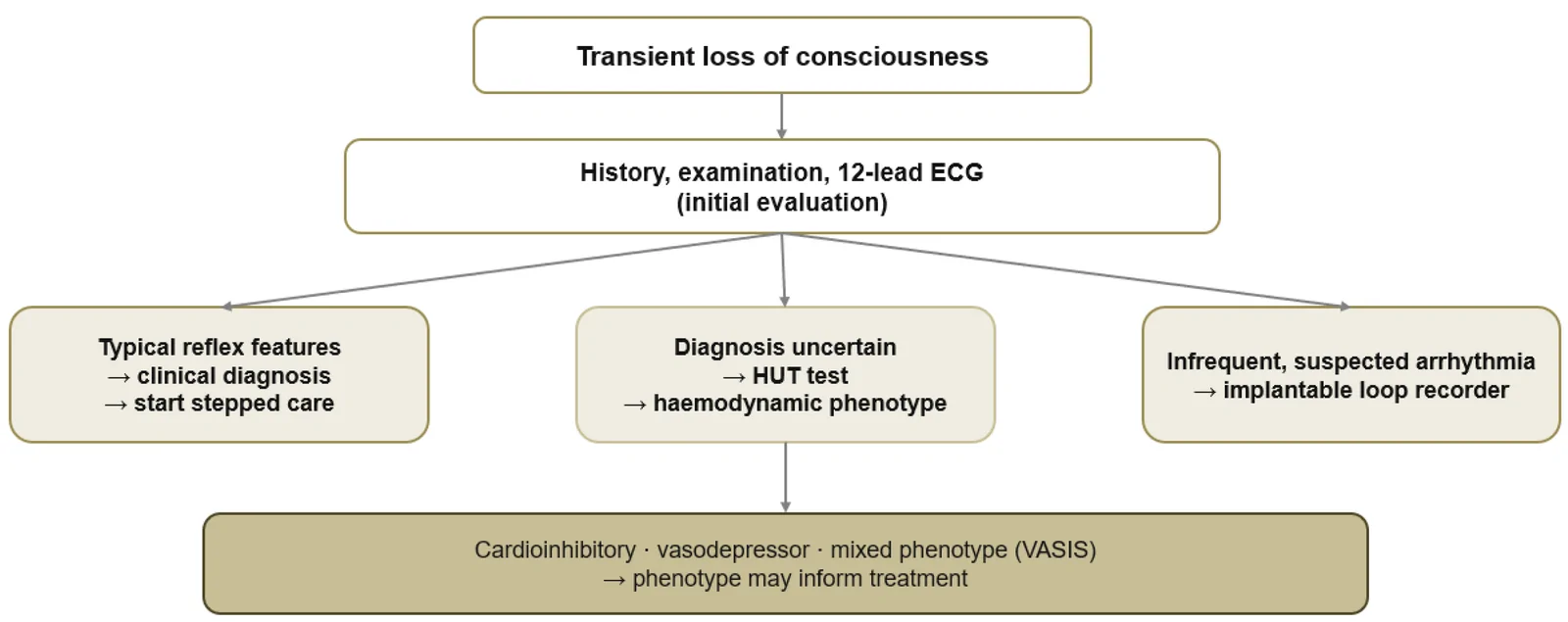
One pragmatic approach integrating the current evidence. After the history, examination, and electrocardiogram, typical cases are managed clinically, uncertain cases proceed to tilt testing and haemodynamic phenotyping, and infrequent cases with a suspected arrhythmia receive an implantable loop recorder, always against the differentials of orthostatic hypotension, postural tachycardia syndrome, and cardiac syncope. Please note that this figure represents the authors’ synthesis of the currently published evidence and guideline recommendations. It is intended as a conceptual framework rather than a formal diagnostic algorithm. ECG, electrocardiogram; HUT, head-up tilt test; and VASIS, Vasovagal Syncope International Study (Brignole et al., 2000) [34].

**Figure 3:**
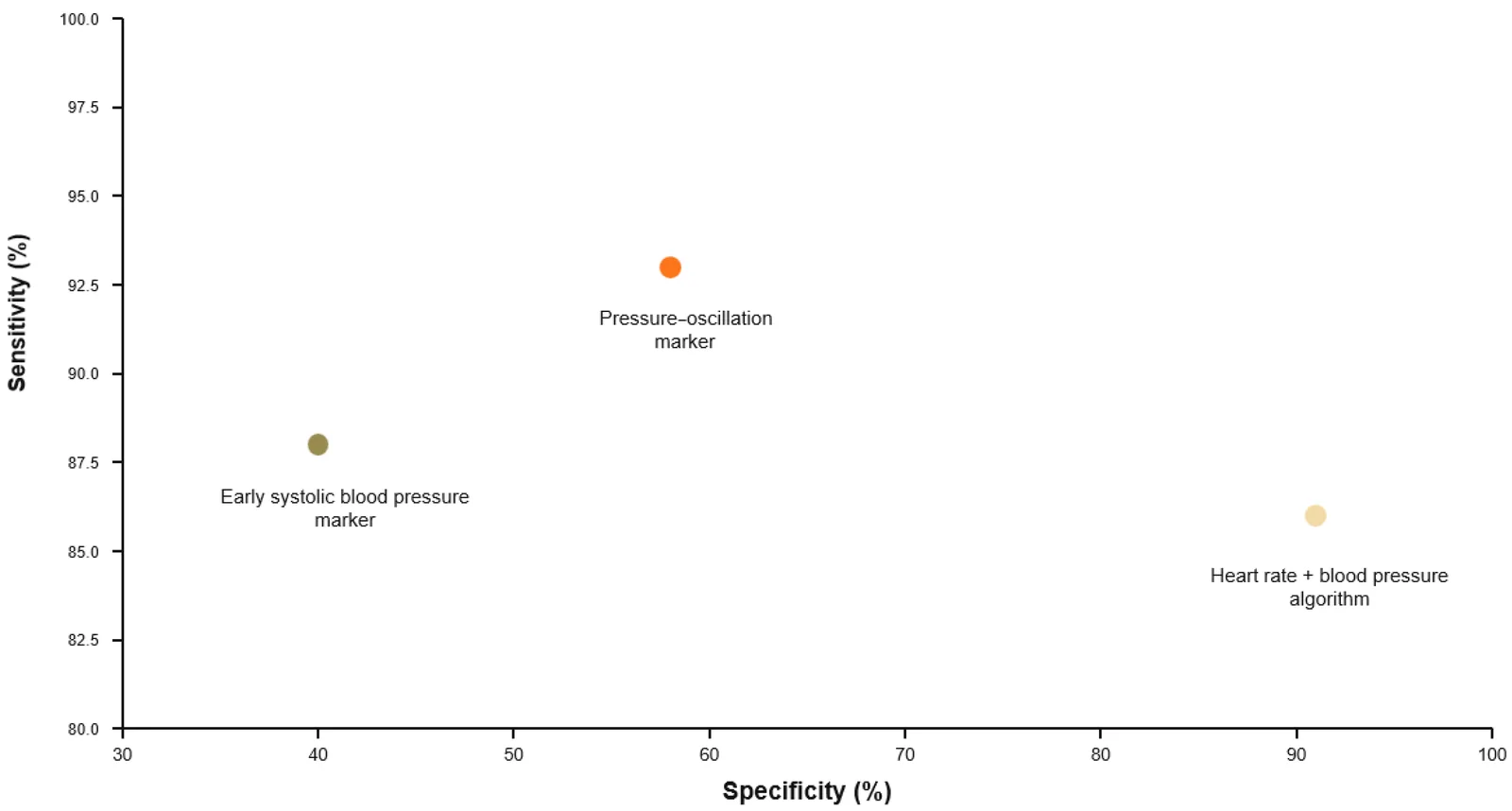
Predicting a positive tilt: sensitivity and specificity of independently published laboratory approaches. Early systolic-pressure and pressure–oscillation markers [48] and a combined heart-rate and blood-pressure algorithm [49] can anticipate tilt-induced syncope. Unpublished quantitative estimates have been removed from the figure.

**Figure 4:**
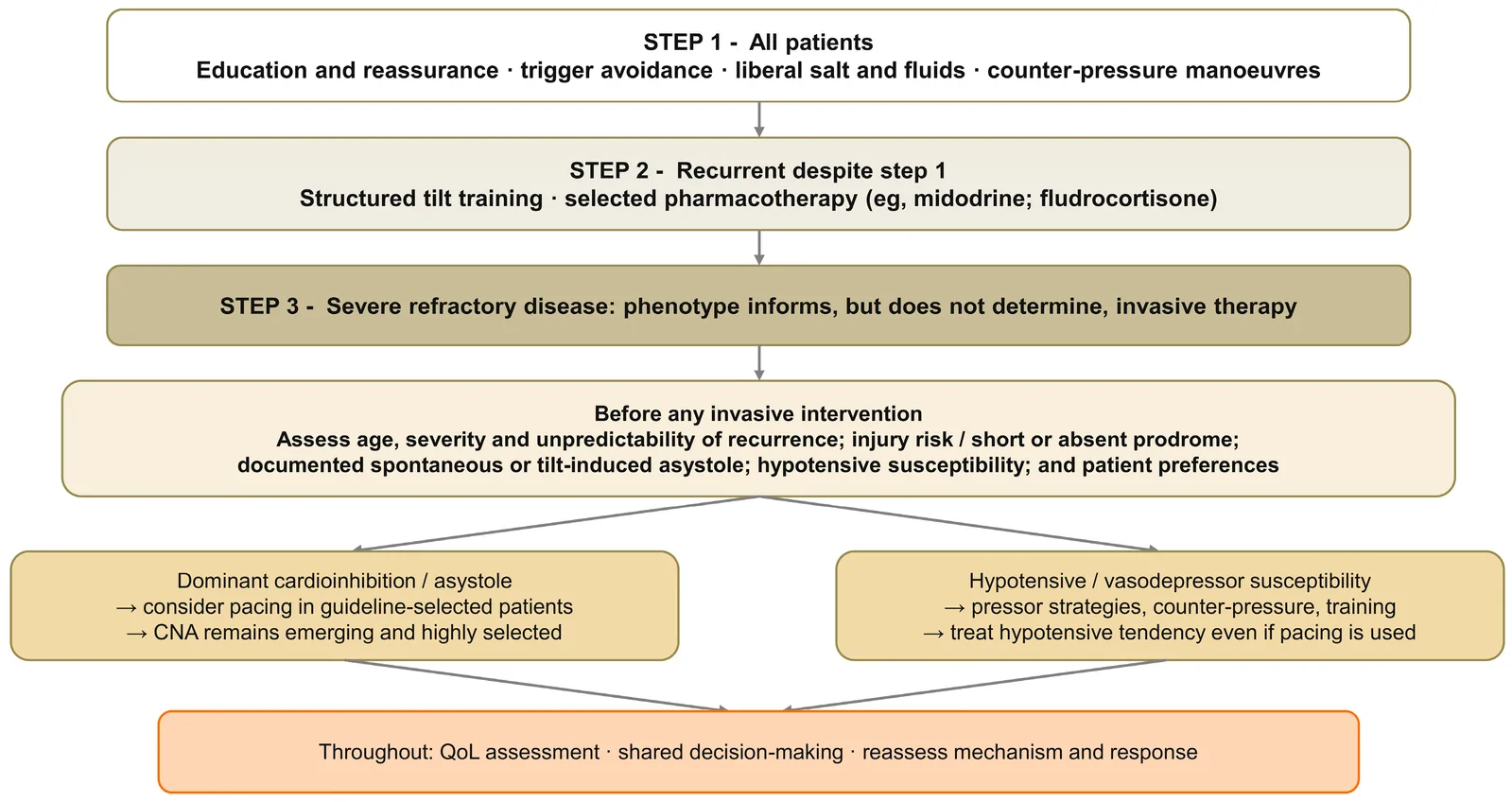
An evidence-informed, phenotype-guided pathway. All patients receive education and counter-pressure manoeuvres; recurrent cases may add tilt training and selected drugs. Before invasive therapy, selection incorporates age, severity, and unpredictability of recurrence, injury risk or short/absent prodrome, documented spontaneous or tilt-induced asystole, hypotensive susceptibility, and patient preferences. The phenotype informs but does not determine the intervention. CNA, cardioneuroablation; and QoL, quality of life.

**Figure 5:**
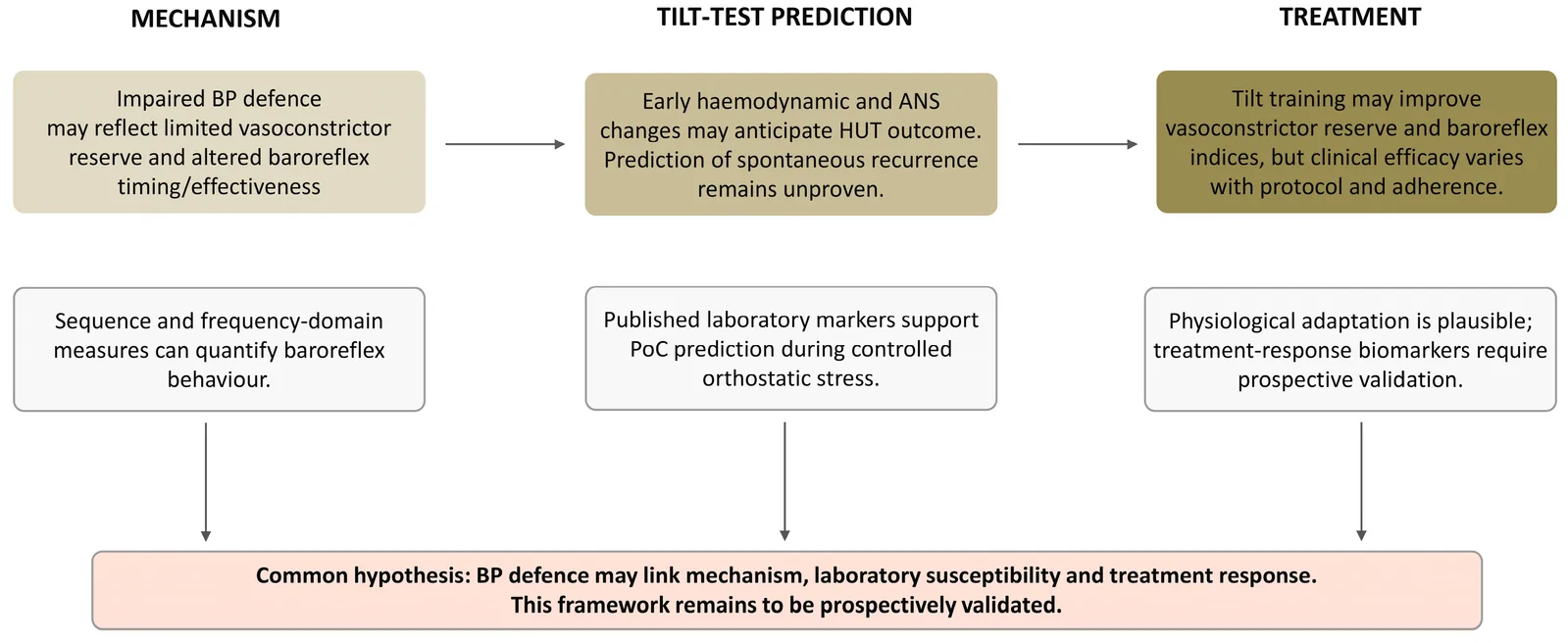
An integrative physiological framework. Arterial-pressure defence and baroreflex behaviour may connect the mechanism, laboratory susceptibility during tilt testing, and physiological adaptation to structured orthostatic training. The framework is hypothesis-generating; the prediction of spontaneous recurrence and treatment-response biomarkers require prospective validation. BP, blood pressure; ANS, autonomic nervous system; HUT, head-up tilt; and PoC, proof of concept.

**Figure 6:**
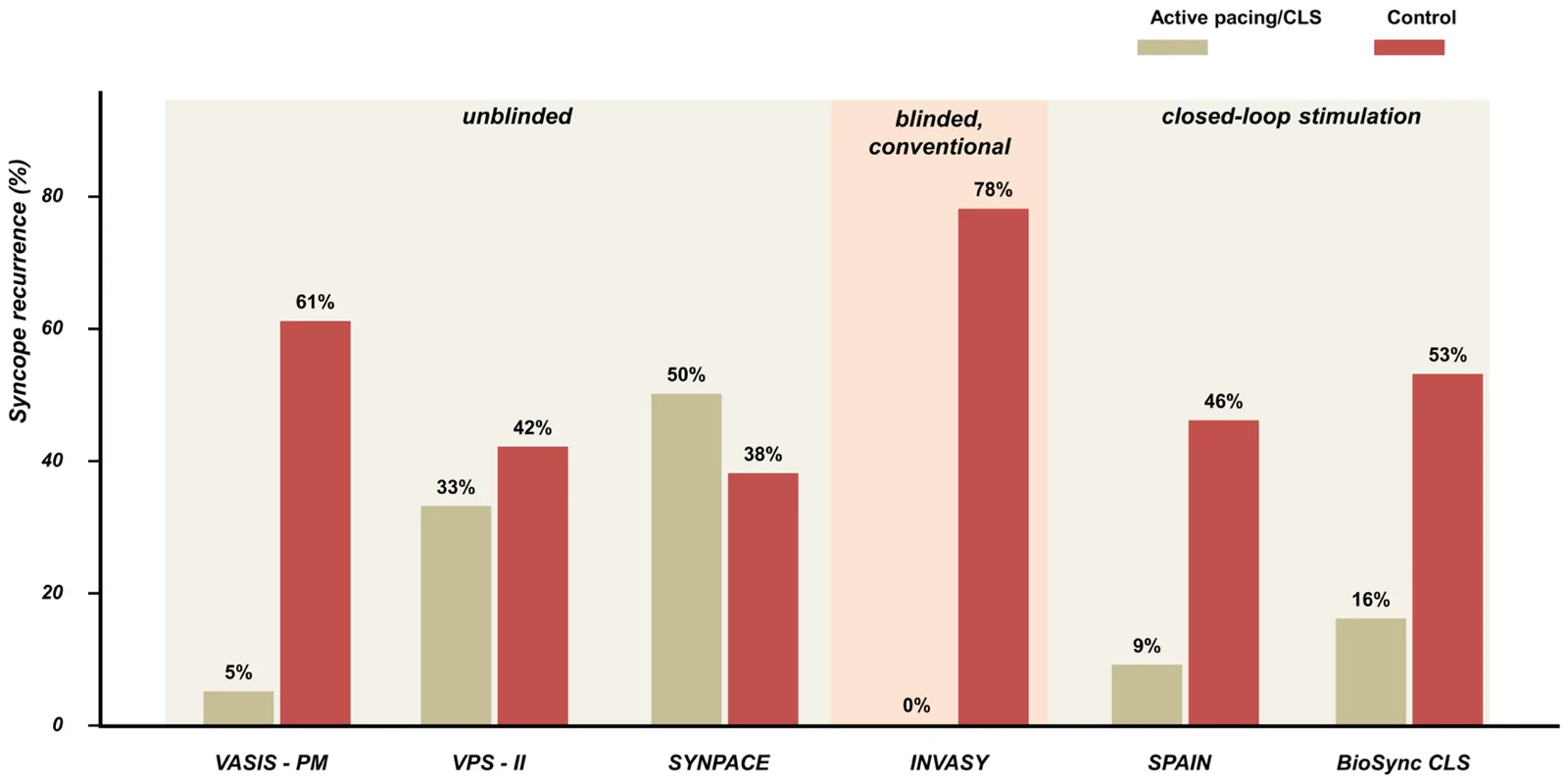
Cardiac pacing for vasovagal syncope. Syncope recurrence with active pacing or closed-loop stimulation and with control across the major trials, grouped by design. Unblinded trials were positive, double-blind trials of conventional pacing were neutral, and only closed-loop stimulation, in selected patients, reduced recurrence under blinding. CLS, closed-loop stimulation; VASIS-PM, VPS II, SYNPACE, INVASY, SPAIN and BioSync CLS, study names as reported in the original publications.

**Figure 7:**
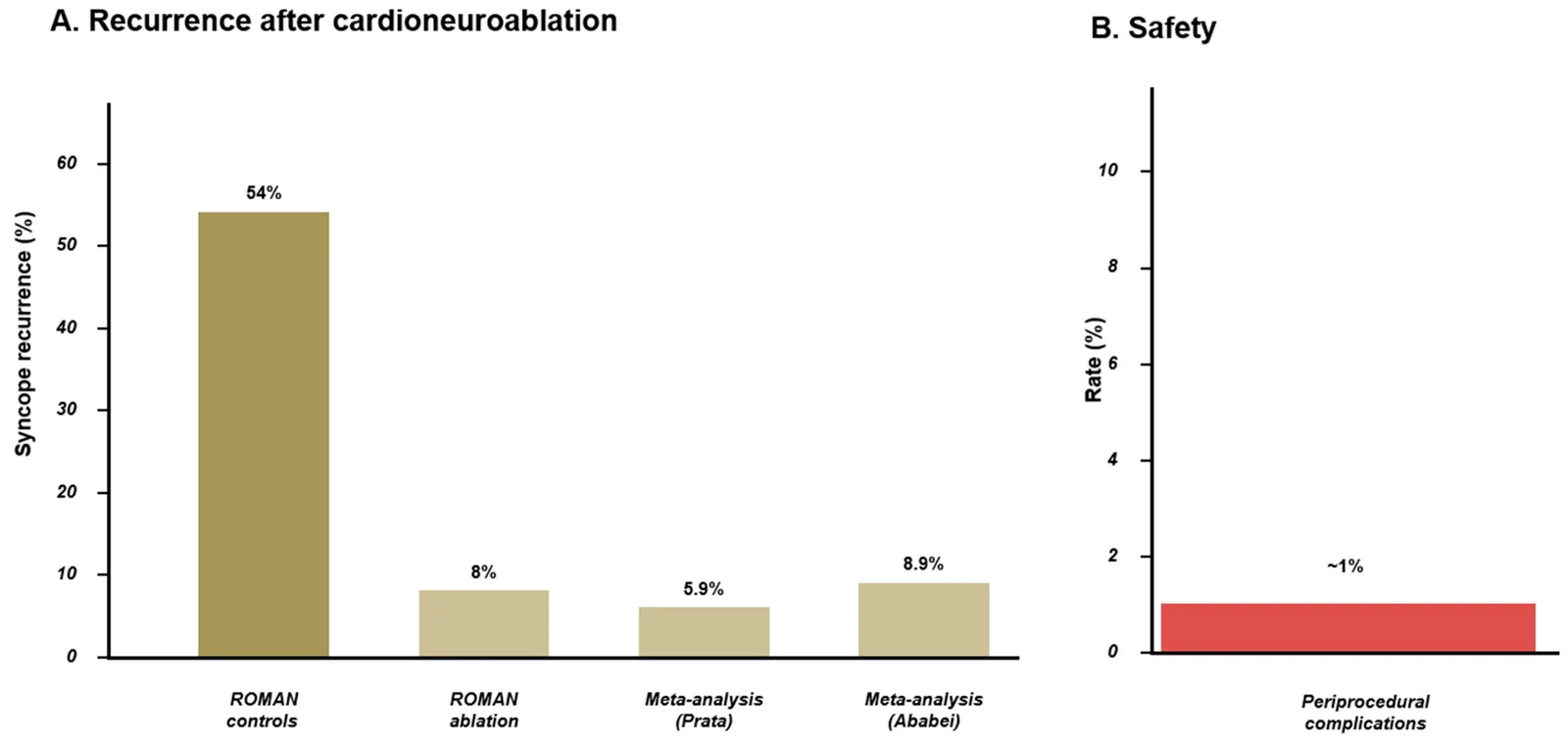
Cardioneuroablation across the evidence. (**A**) Syncope recurrence in the open-label randomised trial and in two meta-analyses [62,63]; and (**B**) the pooled periprocedural complication rate [62]. The estimates should not be interpreted as sham-controlled comparative efficacy. ROMAN, study name as reported in the original publication.

**Figure 8:**
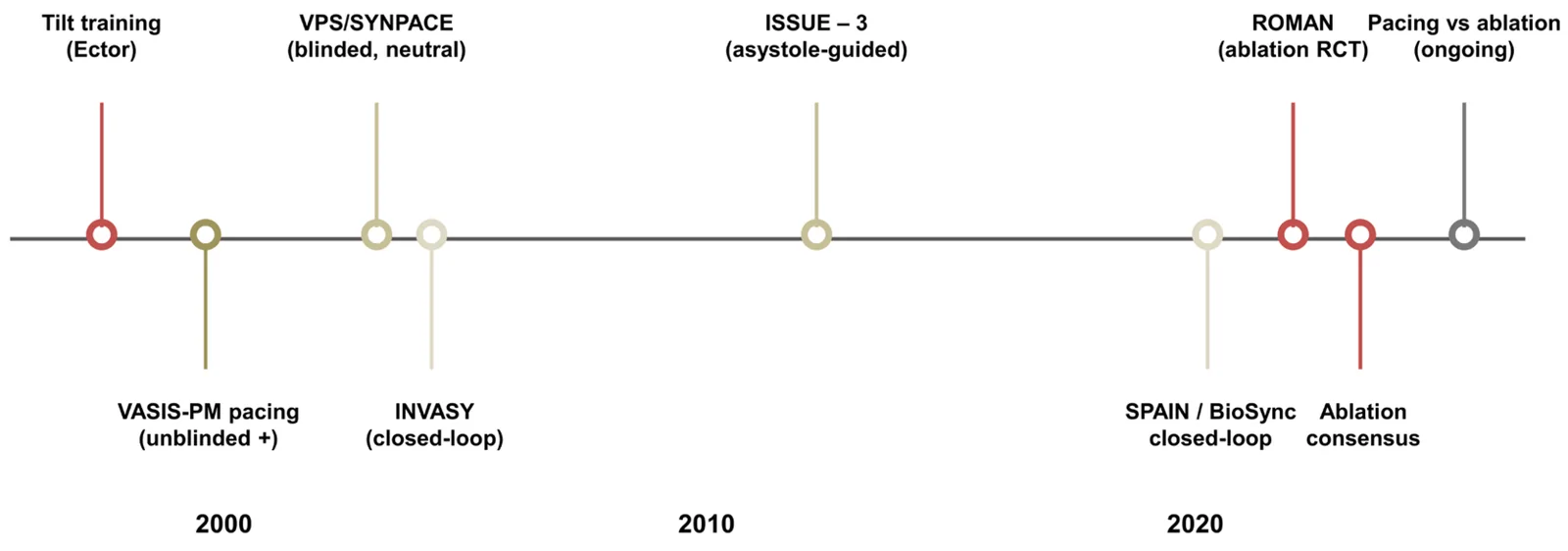
Four decades of evidence, from tilt training to the rise, fall, and selective revival of pacing, cardioneuroablation, and the trials now in progress. RCT, randomised clinical trial; VASIS-PM, VPS II, SYNPACE, INVASY, ISSUE-3, SPAIN, BioSync CLS and ROMAN, study names as reported in the original publications.

**Figure 9:**
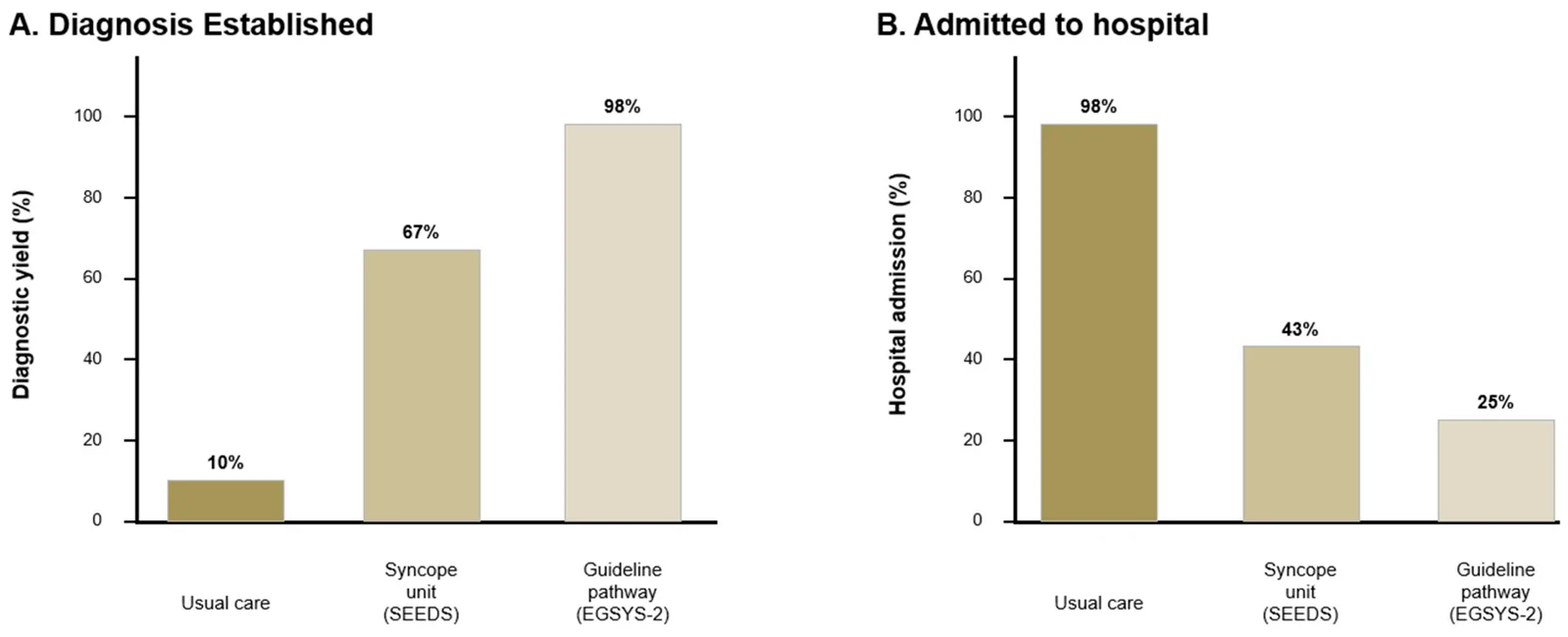
The effect of organised, standardised syncope care on (**A**) the proportion of patients in whom a diagnosis is established [91,92], and (**B**) the proportion admitted to hospital, comparing usual care with a randomised syncope unit and a guideline-based pathway [91,92]. EGSYS-2, Evaluation of Guidelines in Syncope Study 2; and SEEDS, Syncope Evaluation in the Emergency Department Study.

**Table 1:** Conservative and pharmacological treatment of vasovagal syncope (counter-pressure manoeuvres are abbreviated as in the PC-Trial. See the text for the dependence of tilt-training efficacy on adherence) [1,2,51,52,53,54,55,56,57,58].

Intervention	Key Evidence	Comment
Education, salt and fluids	Guideline-based [1,2]	First line for all; low risk
Counter-pressure manoeuvres	Randomised PC-Trial [51]	Aborts or delays the fainting; first-line
Beta-blocker (metoprolol)	POST [52]; POST5 + IPD meta-analysis [58]	No demonstrated reduction in recurrence, including older patients
Fludrocortisone	POST2, randomised [53]	Borderline reduction in recurrence
Midodrine	POST4, randomised [54]	Effective in selected patients; supports vascular tone
Tilt (orthostatic) training	Meta-analysis [55,56]	Non-invasive; efficacy varies with protocol and adherence

IPD, individual patient data; PC-Trial, Physical Counterpressure Manoeuvres Trial; POST, Prevention of Syncope Trial; POST2, POST4, and POST5, study names as reported in the original publications.

**Table 2:** Device and ablation therapies for refractory cardioinhibitory vasovagal syncope. None of these therapies addresses the vasodepressor limb that predominates in younger patients [59,60,61,62,63,64,65,66,67].

Therapy	Key Evidence	Target and Caveats
Dual-chamber pacing	ISSUE-3 [59]; ESC pacing guideline [67]	Highly selected >40-year-old patients with severe recurrent unpredictable syncope and documented asystole
Closed-loop stimulation pacing	SPAIN; BioSync CLS [60,61]	Evidence strongest with tilt-induced asystole; assess hypotensive susceptibility and lifetime device burden
Cardioneuroablation	Open-label randomised trial [62]; Meta-analysis [63,64]	Emerging option; no sham-controlled efficacy trial; long-term effects uncertain
Cardioneuroablation strategy	Left vs. biatrial randomised [65]	Procedural strategy evidence; not comparative efficacy vs. pacing
Multi-society guidance	Scientific statement [66]	Careful patient selection; evidence gaps and long-term autonomic effects remain

CLS, closed-loop stimulation; ESC, European Society of Cardiology; ISSUE-3, Third International Study on Syncope of Uncertain Etiology; SPAIN and BioSync CLS, study names as reported in the original publications.

**Table 3:** Selected ongoing trials and registries (ClinicalTrials.gov, 2026). Identifiers are ClinicalTrials.gov registration numbers; status as of 2026.

Identifier	Question	Design/Status
NCT05855603	Pacing vs. cardioneuroablation for recurrent cardioinhibitory syncope	Randomised, multicentre, recruiting
NCT06440291	Effect of cardioneuroablation on exercise performance (ROMAN 3)	Randomised, recruiting
NCT06159764	National registry of recurrent reflex syncope (CANVAS–R)	Observational registry, recruiting
NCT06166277	Cardioneuroablation for syncope and symptomatic bradyarrhythmia (CNA-FWRD)	Observational registry, recruiting

CNA-FWRD and CANVAS-R, acronyms used in the registered study titles; NCT, ClinicalTrials.gov identifier; ROMAN 3, study name as registered.

**Table 4:** The proposed phenotype-guided approach (hypothesis). This table is a hypothesis-generating framework, not a validated algorithm; see the text. The phenotype is defined on the tilt test using the Vasovagal Syncope International Study scheme [34].

Haemodynamic Phenotype	Dominant Mechanism	Treatment Emphasis (Hypothesis)
Cardioinhibitory/asystolic	Vagal bradycardia/asystole	Phenotype alone is insufficient. If severe recurrent unpredictable syncope with documented clinically relevant asystole consider pacing; cardioneuroablation remains emerging
Vasodepressor	Failure of vasoconstriction/hypotensive susceptibility	Counter-pressure, midodrine, tilt training; pacing is not expected to correct a pure vasodepressor mechanism
Mixed	Both limbs	Address hypotensive susceptibility first; invasive therapy only when clinically relevant cardio-inhibition/asystole is documented and overall selection criteria are met

**Table 5:** The evidence for organised (syncope-unit) care. The syncope unit concentrates and standardises the assessment of the transient loss of consciousness, raising diagnostic yield and reducing admissions and cost [90,91,92].

Source	Intervention	Main Finding
SEEDS [91]	Randomised emergency-department syncope unit	Diagnosis 67% vs. 10%; admission 43% vs. 98%
EGSYS-2 [92]	Guideline-based standardised pathway, 11 hospitals	Diagnosis in about 98%; admission about 25%
EHRA position [90]	Defines rationale, staffing and facilities of a syncope unit	Consensus organisational standard

EGSYS-2, Evaluation of Guidelines in Syncope Study 2; EHRA, European Heart Rhythm Association; SEEDS, Syncope Evaluation in the Emergency Department Study.

## Data Availability

No new data were created or analyzed in this study. Data sharing is not applicable to this article.
